# Network Toxicology and Molecular Docking to Elucidate the Mechanisms of Intestinal Toxicity Induced by P-Phenylenediamine Antioxidants and Their Quinone Derivatives

**DOI:** 10.3390/toxics13060480

**Published:** 2025-06-06

**Authors:** Hui Zou, Yumei Tan, Xiyi Ren, Zhu Li, Yongxiang Liu

**Affiliations:** 1 Key Laboratory of Plant Resource Conservation and Germplasm Innovation in Mountainous Region (Ministry of Education), Collaborative Innovation Center for Mountain Ecology and Agro-Bioengineering (CICMEAB), College of Life Sciences/Institute of Agro-Bioengineering, Guizhou University, Guiyang 550025, China; zouhuimiky@163.com; 2 Guizhou Key Laboratory of Agricultural Microbiology, Guizhou Academy of Agricultural Sciences, Guiyang 550009, China; tanyumei2008@126.com (Y.T.); renxiyiyx@163.com (X.R.)

**Keywords:** network toxicology, P-phenylenediamine antioxidants, intestinal toxicity, molecular docking

## Abstract

P-phenylenediamines (PPDs) and their quinone derivatives (PPDQs), emerging pollutants widespread in urban environments, exhibit biotoxicological risks. Epidemiological studies suggest their adverse impacts on intestinal health, yet the underlying mechanisms remain unclear. This study aimed to investigate the potential mechanisms of enterotoxicity induced by 13 PPDs and PPDQs using network toxicology and molecular docking approaches. Through the SuperPred, STITCH, GeneCards, and OMIM databases, 182 potential targets associated with PPD- and PPDQ-induced enterotoxicity were identified. Thirty hub targets, including SRC, EGFR, CASP3, and others, were prioritized using STRING and Cytoscape tools. GO and KEGG enrichment analyses via the DAVID and FUMA databases revealed significant enrichment of core enterotoxicity-related targets in the MAPK signaling pathway and the calcium signaling pathway. Molecular docking with AutoDock confirmed strong binding affinities between PPDs/PPDQs and core targets. These results suggest that PPDs and PPDQs may promote the onset and progression of bowel cancer and intestinal inflammation by modulating cancer cell death, proliferation, and inflammatory signaling pathways. This research provides a theoretical framework for elucidating the molecular mechanisms of PPD- and PPDQ-induced enterotoxicity, offering insights for the prevention of associated diseases.

## 1. Introduction

P-phenylenediamines (PPDs), a class of synthetic antioxidants, are widely recognized for their superior thermal stability, resistance to degradation, and protection against ozone-induced cracking. Common p-phenylenediamine antioxidants include 6PPD, N-phenyl-N′-cyclohexyl-p-phenylenediamine (CPPD), N, N’-bis (1,4-dimethylpentyl)-p-phenylenediamine (77PD), N-isopropyl-N′-phenyl-1,4-phenylenediamine (IPPD), N, N′-di-β-naphthyl-p-phenylenediamine (DNPD), and N, N′-diphenyl-p-phenylenediamine (DPPD). They are widely used in the production of rubber products such as tape hoses and automotive tires as well as in a variety of everyday consumer products, such as apparel, recreational facilities, and sports equipment [1]. However, the abrasion of rubber tires releases PPDs into the urban environment along with tire wear particles. Once released into the environment, PPDs can undergo transformation into PPD quinones (PPDQs) through multiple chemical pathways, including 6PPD-Q, 2-(cyclohexylamine)-5-(phenyl-amino)cyclohexa-2,5-diene-1,4-dione (CPPD-Q), and 2,5-bis(phenylamino)cyclohexa-2,5-diene-1,4-dione (DPPD-Q). Moreover, extensive evidence demonstrates the widespread presence of PPDs and PPDQs in urban runoff, sediments, and air [2,3] and also in human blood, urine, and breast milk [4]. Due to their extensive presence in numerous facets of the daily life of mankind, it is essential to examine the harmful consequences of PPDs and PPDQs.

To date, studies have demonstrated that PPDs and PPDQs are biotoxic, causing harm to growth, development, and reproduction as well as neurotoxicity and enterotoxicity [5,6]. Regarding developmental toxicity, 6PPD-Q has been shown to induce acute lethality in coho salmon (*Oncorhynchus kisutch*) [7]. Additionally, IPPD and 6PPD have been reported to cause motor dysfunction in zebrafish (*Danio rerio*) embryos, larvae, and adults while also inhibiting their growth and development [8]. In terms of reproductive toxicity, studies have demonstrated that exposure to 1–10 μg/L of 6PPD-Q from the L1 larval stage for 4.5 days significantly impairs the reproductive capacity of *C. elegans*, manifesting as reduced egg counts in the uterus and decreased hatching rates [9]. Concerning neurotoxicity, 6PPD-Q has been found to activate neutrophils and induce overexpression in enteric neurons. Furthermore, animal studies indicate that 6PPD-Q accumulates in the brain. Researchers have investigated the presence of this compound in the cerebrospinal fluid (CSF) of Parkinson’s disease (PD) patients and healthy controls, revealing significantly higher levels in PD patients compared to the control group [10]. Among them, the intestinal tract is one of the most important organs in the human body, undertaking multiple vital physiological functions, including food digestion and absorption, immune regulation, and barrier defense. However, the invasion of toxic substances such as environmental pollutants can severely affect the function of the gut and even disrupt its barrier function, leading to chronic inflammation, immune disorders, and other health problems. Globally, the incidence of intestinal cancers is on the rise, especially colon and rectal cancers. According to forecasts, the total number of deaths from colon and rectal cancer will increase by 71.5% and 60%, respectively, by 2035 [11]. Therefore, among the multiple biotoxicities induced by PPDs and PPDQs, we focus on their toxic effects on the gut. According to recent research, 6PPD-Q in PPDQs may cause intestinal toxicity. Altered intestinal anatomy and increased blood coagulability were observed in zebrafish larvae after exposure to 6PPD-Q [12]. Additionally, some PPDQs have been shown to induce intestinal toxicity in *Caenorhabditis elegans*, disrupting the intestinal barrier and causing intestinal oxidative stress [13]. Previous studies have focused on the enterotoxicity of common PPD derivatives (e.g., 6PPD-Q and DPPD). However, systematic studies on the enterotoxicity of other PPDs and PPDQ contaminants are still limited. Furthermore, the molecular mechanisms linking PPDs and PPDQ exposure to intestinal toxicity remain unclear. Therefore, additional research is essential to comprehensively elucidate the impact of PPDs and PPDQs on intestinal health.

Considering that various biomolecular networks can be disrupted by environmental pollutants, which results in complicated toxicities, it is necessary to develop novel methods for comprehensive toxicological evaluation. It is challenging to thoroughly and successfully evaluate the possible biological impacts of these environmental contaminants by conventional toxicological detection methods because conventional toxicological research methodologies sometimes only concentrate on a one-sided “one target, one drug” model with poor correlation between toxic pathways [14]. Therefore, to properly evaluate the health risks of environmental contaminants, creative and methodical approaches are required.

Combining network toxicology with molecular docking is a promising strategy [15]. Network toxicology is a systematic research methodology integrating multi-omics data that combines bioinformatics, genomics, proteomics, and metabolomics approaches to construct compound–target–pathway interaction networks. This approach is particularly suitable for deciphering complex toxicity mechanisms involving multiple components and targets, transforming intricate biological information into intuitive network models. Through network topology analysis, this technique can effectively reveal the perturbation patterns of xenobiotic-mediated protein–protein interaction networks, thereby establishing a novel research paradigm for elucidating the molecular mechanisms of toxicant-induced diseases [16]. Molecular docking is a popular virtual screening approach that simulates receptor–ligand binding by calculating parameters to predict binding affinity and mode. Specifically, molecular docking technology simulates and analyzes the spatial conformations and binding characteristics between small molecule compounds and proteins at the atomic level. This method holds unique value in toxicological research, as it enables the prediction of specific binding interactions between toxins and biomacromolecules, thereby providing theoretical foundations for elucidating toxicity mechanisms and potential biological damages [17]. The integrated application of network toxicology and molecular docking has demonstrated significant value in drug safety assessment and environmental pollutant toxicity screening, establishing itself as a core methodological combination in modern computational toxicology research [18]. With this strategy, recent studies have highlighted the molecular mechanisms involved in cancer and liver damage caused by the environmental pollutant thiabendazole [19].

This study integrates network toxicology and molecular docking approaches to analyze the toxicological effects associated with 15 PPDs and PPDQs in inducing enterotoxicity. The aim is to elucidate their toxicological mechanisms, predict potential toxicity, clarify their threat to human intestinal health, and provide a reference for studying related diseases caused by exposure to these toxic compounds.

## 2. Methods

### 2.1. Target Construction of PPDs and PPDQs

Thirteen p-phenylenediamine antioxidants and their quinone derivatives (PPDs/PPDQs) were systematically retrieved from the PubChem database, including 6PPD, 77PD, CPPD, DPPD, DTPD, 7PPD, DNPD, IPPD, 44PD, and common quinone compounds (6PPD-Q, CPPD-Q, DPPD-Q, and IPPD-Q). The two-dimensional structural information for all compounds was downloaded and stored in SDF file format. The SDF files were subsequently submitted to the SwissTargetPrediction platform (http://www.swisstargetprediction.ch/; accessed on 10 November 2024) and the prediction condition probability > 0 was set. The prediction results were analyzed in Excel, retaining only targets meeting this probability criterion as potential binding sites for PPDs and PPDQs. Then, the SDF files were submitted to the SuperPred web server (https://prediction.charite.de/; accessed on 12 November 2024) for analysis, the prediction condition probability ≥ 50 was set, and the prediction results were submitted and downloaded. Following data integration by merging the target lists and removing duplicate entries, a comprehensive target database for PPDs and PPDQs was ultimately established.

### 2.2. Target Construction of Intestinal Toxicity

The OMIM (https://www.omim.org/; accessed on 15 November 2024) and GeneCards (https://www.genecards.org/; accessed on 20 November 2024) databases were utilized to create disease–target libraries. Briefly, the keyword “intestinal toxicity” was used to search for targets in the two databases already mentioned. Because GeneCards predicted more targets, the top targets (5000) were filtered, respectively. After merging the expected targets from the two databases and removing duplicates, the resulting targets were used for subsequent analysis.

### 2.3. Acquisition of Intersection Targets

The common targets among PPDs, PPDQs, and intestinal toxicity were identified by crosswalking their target sets. A Venn diagram was generated using the Venny2.1.0 online platform (https://bioinfogp.cnb.csic.es/tools/venny/; accessed on 22 November 2024) to visualize the overlapping targets.

### 2.4. Constructing of PPI Network

The intersected genes, which indicate potential targets of PPDs- and PPDQ-induced intestinal toxicity, were introduced into the STRING database (https://cn.string-db.org/; accessed on 23 November 2024), and the species parameter was set to “*Homo sapiens*”, setting the “Minimum required interaction score” to “Medium Confidence > 0.4” to generate the interaction network. Subsequently, the TSV file containing core (hub) target data was downloaded, and network topology and core node identification analyses were conducted using Cytoscape software (version 3.10.0). The screened core targets must simultaneously meet the following three criteria based on the network graph analysis of gene nodes using the Centiscape plugin for core target screening, which calculates the closeness centrality, betweenness centrality, and degree value of each node: (1) closeness centrality > median; (2) betweenness centrality > median; (3) degree value > median.

### 2.5. Constructing of Drug–Target–Pathway Network

The initial step involved creating a feature table in Excel, which lists the primary targets associated with the compounds along with all relevant biological pathways linked to each target. Subsequently, the data were imported into Cytoscape by loading the feature table through the Network file interface to establish node positions. The visualization was then adjusted by defining the origin point and altering the graphical representations.

### 2.6. GO and KEGG Pathway Analysis

To identify functional annotations and pathway enrichment associated with putative genes, the GO and KEGG pathways were analyzed in the DAVID online tool. The top 20 KEGG pathways and the top 10 GO terms were chosen according to the ascending order of the *p*-values. Subsequently, the Bioinformatics online platform was utilized to perform visual analysis, enabling the interpretation and visualization of the KEGG and GO analysis results.

### 2.7. Molecular Docking

The crystal structures of the key proteins were obtained from the RCSB Protein Data Bank (PDB). When multiple structures of the same target protein were available, the selection criteria were as follows: The structure with the highest resolution that covers either the small-molecule docking site or the full-length protein was selected from human-derived proteins based on literature evidence. All protein structures were rigorously prepared prior to analysis. Then, water molecules and small-molecule ligands were removed from the protein structures prior to docking analysis through PyMOL software (version 2.5.2). Using AutoDockTools (version 1.5.7), the docking grid points and dimensions were optimized to ensure complete coverage of the protein within the docking box. Semi-flexible molecule docking simulations were carried out using AutoDock Vina software (version 1.2.3). A higher absolute value of binding energy (ΔG) indicates stronger binding affinity between the receptor and the ligand.

## 3. Results

### 3.1. Potential Targets of PPDs- and PPDQs-Induced Intestinal Toxicity

**Figure 1:**
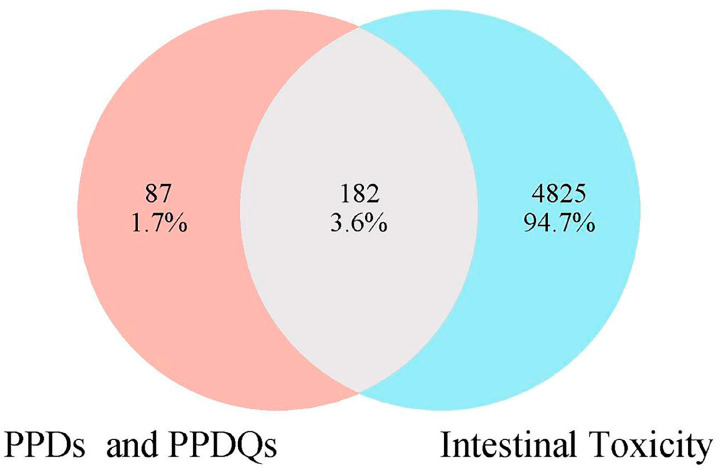
The overlapped targets of PPDs, PPDQs, and intestinal toxicity.

In this study, 269 PPDs and PPDQs targets were initially sifted from the Swiss Target Prediction and SuperPred databases. Then, a total of 5007 intestinal toxicity targets were gathered utilizing the GeneCards and OMIM databases. PPDs and PPDQs targets and enterotoxin-related targets were input into Venny2.1 for intersection, and 182 intersected targets were identified (Figure 1), serving as potential targets for intestinal toxicity caused by PPDs and PPDQs.

### 3.2. PPI Network and Key Target Acquisition

**Figure 2:**
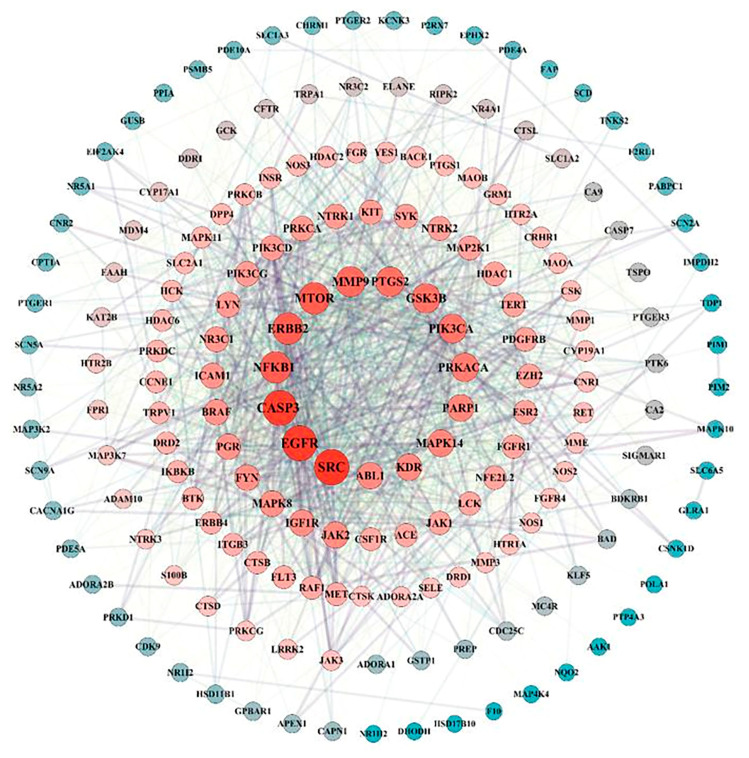
PPI network of intersected targets.

**Figure 3:**
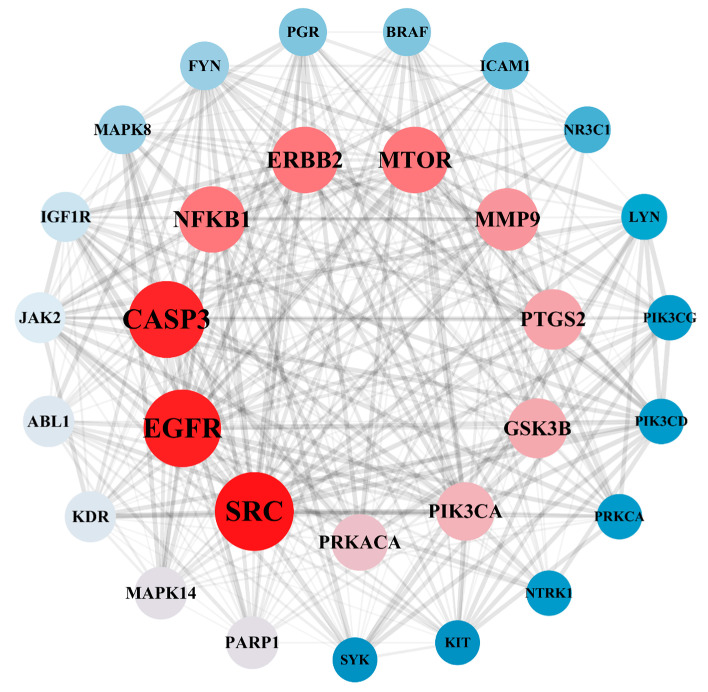
PPI network of hub targets.

A total of 182 intersection targets were imported into the STRING database for analysis and PPI network construction, resulting in a network of 78 nodes and 334 edges. The topological properties of the network nodes, including closeness, betweenness, and degree, were analyzed using Cytoscape software (version 3.10.0). Additionally, a visually optimized protein–protein interaction network diagram was generated, as shown in Figure 2. Through further network analysis, 30 core targets of PPD- and PPDQ-induced intestinal toxicity were ultimately identified (Table 1), and a protein–protein interaction (PPI) network was constructed (Figure 3) to specifically characterize the interactions among these core targets. Based on their degree values, the top five core targets are non-receptor tyrosine kinase (SRC), epidermal growth factor receptor (EGFR), caspase 3 (CASP3), serine/threonine protein kinase (MTOR), and tyrosine kinase receptor 2 (ERBB2). The proteins encoded by the above genes are critical for a variety of cellular processes, such as immune response regulation, cell cycle control, apoptosis, and other processes, all closely linked to cell growth, development, and tumorigenesis.

### 3.3. Target Function Analysis and Pathway Enrichment Analysis

**Figure 4:**
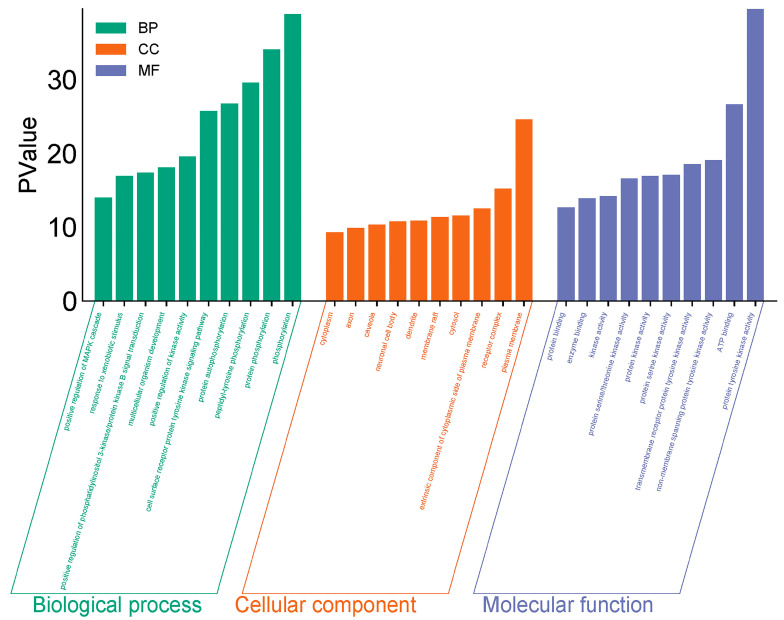
GO diagram of PPDs and PPDQs induced intestinal toxicity.

**Figure 5:**
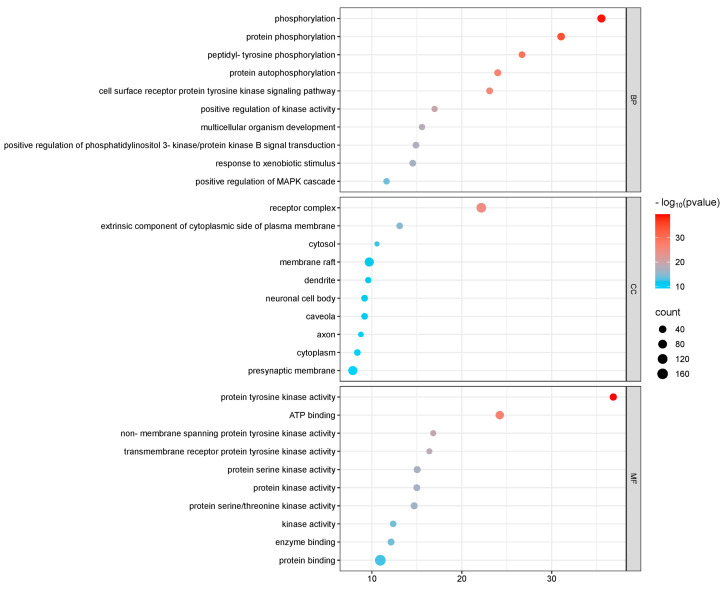
GO *p*-value diagram of PPDs and PPDQs.

GO and KEGG enrichment analyses were conducted on 182 intersection targets utilizing the DAVID database, selecting species parameters as “*Homo sapiens*”. The GO enrichment analysis yielded 850 items, consisting of 607 biological processes (BP), 95 cellular components (CC), and 148 molecular functions (MF) (Figure 4). The biological processes identified include phosphorylation, protein phosphorylation, and peptide tyrosine phosphorylation within the cell surface receptor protein tyrosine kinase signaling pathway. The key cellular components involved are the plasma membrane, receptor complexes, exogenous components, and cytoplasmic components associated with the plasma membrane. Notable molecular functions include protein tyrosine kinase activity, ATP-binding protein binding, non-transmembrane protein tyrosine kinase activity, and transmembrane receptor protein tyrosine kinase activity (Figure 5).

**Figure 6:**
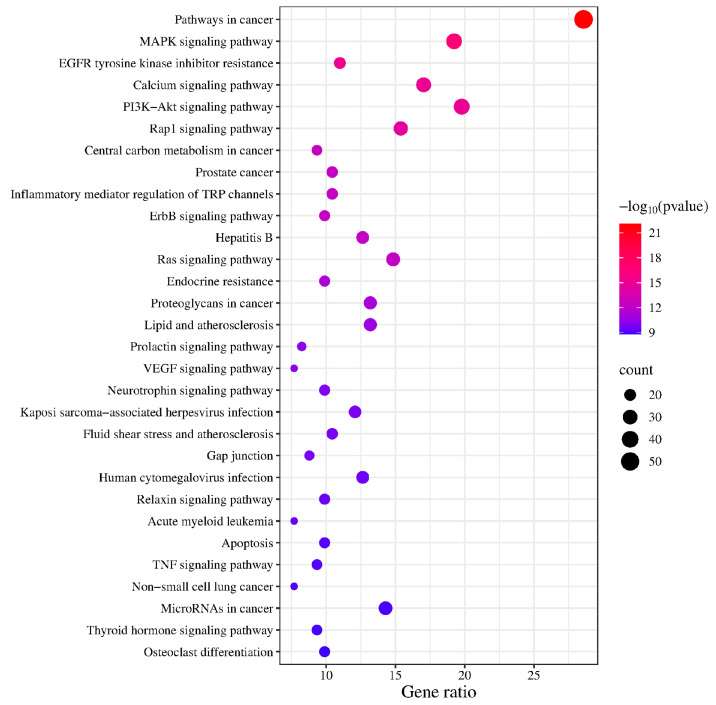
The top 20 KEGG pathways.

**Figure 7:**
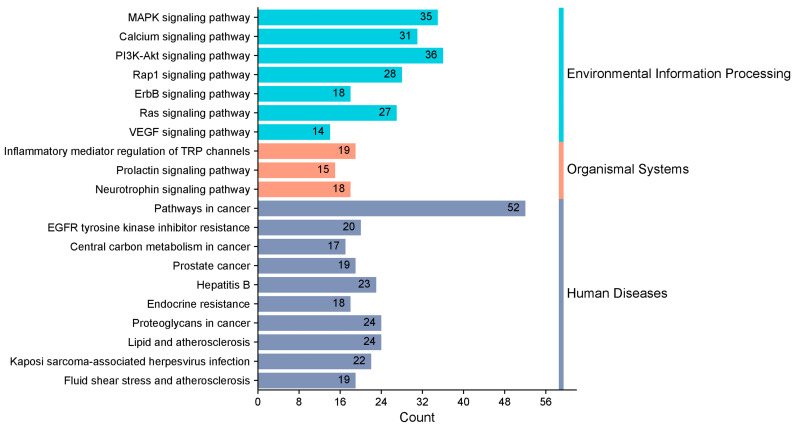
Histogram depicting the categorization of KEGG pathways and the frequency of enrichment.

KEGG enrichment analysis identified 163 pathways, among which the top 20 pathways were chosen for visualization with the lowest *p*-values. The number of genes is indicated by the size of the circles; larger circles indicate more genes. To further illustrate the number of genes enriched, a bar graph was also created (Figure 6). The top four pathways with the highest gene enrichment were the cancer pathway (52 genes), the MAPK signaling pathway (36 genes), the epidermal growth factor receptor tyrosine kinase inhibitor resistance pathway (35 genes), and the calcium signaling pathway (31 genes) (Figure 7). These results suggest that PPD- and PPDQ-induced enterotoxicity is primarily associated with these four pathways.

### 3.4. Analysis of the Intestinal Toxicity Network Induced by PPDs and PPDQs

**Figure 8:**
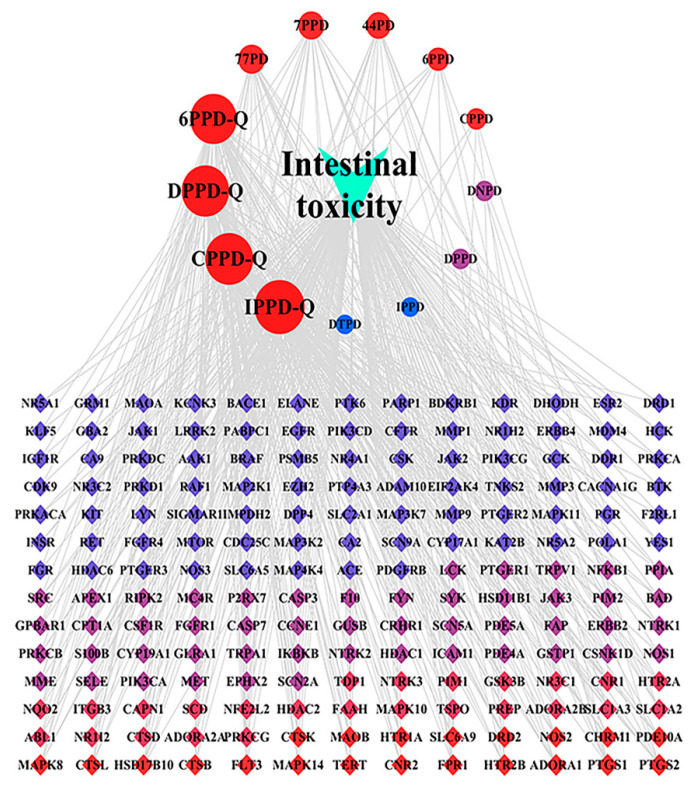
“Drug–target–pathway” network of PPDs and PPDQs induced intestinal toxicity.

The “drug–target–pathway” network illustrating the intestinal toxicity induced by PPDs and PPDQs was created using Cytoscape. The hub targets, related pathways, and the degree of intestinal toxicity caused by PPDs and PPDQs are all shown in this diagram (Figure 8). It consists of 196 nodes and 533 edges. Notably, a single active ingredient can correspond to multiple targets, and different active ingredients may share the same target. This illustrates the multi-target nature of the pollutant effects on diseases. The top five nodes, ranked by degree value, corresponded to IPPD-Q, CPPD-Q, DPPD-Q, 6PPD-Q, and 77PD. Among them, the highest degree is IPPD-Q (Table 2).

### 3.5. Molecular Docking for IPPD-Q and Core Target Proteins of Intestinal Toxicity

**Figure 9:**
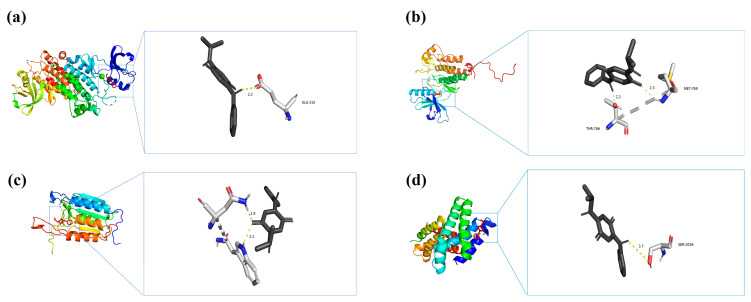
Molecular docking results of each hub target protein with IPPD-Q. (**a**) IPPD-Q–SRC, (**b**) IPPD-Q–EGFR, (**c**) IPPD-Q–CASP3, and (**d**) IPPD-Q–MTOR.

According to their degree centrality in the PPI network, the top four key genes (SRC, EGFR, CASP3, and MTOR) were selected for molecular docking analysis with IPPD-Q (Table 3), the most representative chemical component of PPDs and PPDQs. All four docking results were generated using AutoDock software (version 1.5.7) and showed low binding energies. The binding energies of IPPD-Q to MTOR, SRC, EGFR, and CASP3 were −6.8, −6.7, −6.7, and −6.1 kcal·mol^−1^, respectively. Noticeably, the four core target proteins exhibited robust binding to IPPD-Q and all binding energies of less than −5 kcal·mol^−1^, suggesting IPPD-Q can bind spontaneously to the four key target proteins (Figure 9), indicating their important roles in the molecular mechanism underlying PPDs and PPDQs-induced intestinal toxicity.

## 4. Discussion

In this study, 182 potential targets related to the intestinal toxicity induced by PPDs and PPDQs were systematically screened using the STITCH, SuperPred, GeneCards, and OMIM databases. A network was created utilizing the STRING platform and Cytoscape, and 30 key targets were identified, including SRC, EGFR, CASP3, MTOR, and ERBB2. These targets represent the core components in the context of intestinal toxicity induced by PPDs and PPDQs.

SRC is a non-receptor tyrosine-protein kinase located in cell membranes and encoded by the proto-oncogene SRC [20]. Previous studies have detected abnormal SRC expression in cells of diseased intestinal tissue in various conditions, including ulcerative colitis (UC) and bowel cancer [21,22]. The SRC protein is linked to numerous malignancies, according to the KEGG pathway analysis results, which is consistent with previous studies. It is essential to the progression of cancer, for example, through the promotion of cancer cell proliferation, remodeling of the cancer cell cytoskeleton, and triggering the invasion and metastasis of growth mechanisms [23]. Notably, approximately 80% of colorectal cancer (CRC) specimens exhibit significantly higher SRC expression compared to normal colonic epithelium, with metastatic lesions demonstrating even greater Src activity than primary tumors. Studies have revealed that SRC activity in CRC tissues is 5- to 8-fold higher than in normal colonic mucosa, while primary colon tumors show 5- to 7-fold increased SRC activity relative to adjacent normal tissue. Dysregulation of c-SRC signaling may cooperate with other oncogenic pathways to drive malignant transformation through uncontrolled proliferation, enhanced cell survival, and increased invasiveness. Upon activation by growth factors or integrins, c-SRC initiates downstream signaling cascades, including the RAS/MAPK, phosphatidylinositol 3-kinase (PI3K)/AKT, and STAT pathways. These events promote aberrant proliferation of intestinal epithelial cells, suppress apoptosis, and ultimately contribute to the development of aggressive cancer phenotypes. Based on these findings, it can be hypothesized that SRC may increase susceptibility to colorectal cancer [24].

EGFR is a member of the ERBB family of cell surface receptor tyrosine kinases and plays a vital role in regulating tumor cell growth, differentiation, and survival [25]. Mutations in EGFR can lead to the continued activation of the kinase even in the absence of a ligand, contributing to its carcinogenic potential. This activation promotes tumor cell proliferation, adhesion, invasion, and metastasis by engaging pathways such as Ras/Raf/ MEK/ERK and PI3K/PDK1/Akt. Consequently, EGFR is necessary for the development and progression of various tumors [26,27]. Previous studies have shown that the average optical density of EGFR-positive expression in colorectal cancer tissues is significantly higher than in normal colorectal mucosal tissues, indicating that the onset and progression of colorectal cancer are significantly influenced by EGFR, and high expression of EGFR can drive bowel cancer progression [28]. Extensive fundamental research has established that EGFR exerts its oncogenic effects through activation of downstream signaling cascades, particularly the RAS-RAF-MAPK and PI3K-AKT-mTOR pathways, which collectively drive aberrant proliferation of intestinal epithelial cells, enhance metastatic potential, and promote tumor angiogenesis [29]. Given EGFR’s pivotal role in colorectal carcinogenesis, multiple classes of EGFR-targeted therapeutics have been successfully translated into clinical applications. Notable examples include monoclonal antibodies such as cetuximab and panitumumab, which specifically target the EGFR pathway [30]. Of particular significance is EGFR’s identification as a core molecular target in PPD/PPDQ-induced intestinal toxicity. This mechanistic understanding provides a rational basis for hypothesizing that PPD and its quinone derivatives may exert intestinal toxic effects through EGFR-mediated pathogenic processes.

CASP3, a cysteine-aspartate protease, is part of the caspase family and a key regulator in the apoptosis and necrosis pathways [31]. Its activation by initial caspases, such as caspase-8, caspase-9, and caspase-10, initiates a series of reactions that lead to cell death [32]. Research indicates that caspase-3 may also facilitate the growth, migration, and invasiveness of cancer cells [33]. Indeed, CASP3 plays a dual regulatory role in both intestinal injury and colorectal cancer development through its modulation of apoptotic and inflammatory processes. Under acute intestinal damage conditions, excessive CASP3 activation induces massive apoptosis of intestinal epithelial cells, disrupts tight junction protein expression, increases intestinal permeability, and triggers inflammatory responses. Conversely, during chronic pathological states, sustained CASP3 activation stimulates compensatory proliferative signaling pathways (including NF-κB and Wnt/β-catenin pathway activation), thereby promoting the initiation and progression of colorectal carcinogenesis [34]. Furthermore, studies have shown that overexpression of CASP3 in colon cancer exacerbates the pathologic progression of the disease. Compared to patients with low CASP3 expression, those with high expression have more severe tumor invasion and metastasis, as well as a shorter survival time [35].

MTOR is an atypical serine-threonine kinase that promotes nutritional metabolism; participates in apoptosis, autophagy, and inflammatory response; and is closely associated with various inflammatory diseases and cancers, mTOR promotes intestinal pathogenesis through hyperactivation of downstream signaling pathways. Persistent activation of mTORC1 inhibits autophagy in intestinal epithelial cells, leading to oxidative stress and apoptosis. Concurrently, it enhances production of pro-inflammatory cytokines (e.g., IL-1β and TNF-α) via S6K1, thereby compromising intestinal barrier integrity and contributing to colorectal carcinogenesis [36]. One study detected high expression of mTOR in 2,4,6-trinitrobenzene sulfonic acid (TNBS)-induced colitis cells in mice, and inhibition of mTOR expression significantly alleviated the symptoms of enteritis, restored the barrier function of the intestinal epithelium, and reduced intestinal damage in mice. In addition, the occurrence of ulcerative colitis (UC) and juvenile intestinal polyposis syndrome (JPS) is also associated with abnormal expression of MTOR. In both diseases, MTOR activity is over-activated or inhibited, thereby disrupting normal intestinal immune regulation [37,38].

Tyrosine kinase receptor-2 (ERBB2) is a member of the epidermal growth factor receptor (EGFR) family. It plays a critical role in biological processes such as cell growth, proliferation, and differentiation. ERBB2 orchestrates intestinal pathogenesis through coordinated mechanisms by constitutively activating downstream PI3K/AKT and RAS/MAPK signaling axes, which concurrently drive malignant proliferation and apoptosis resistance in intestinal epithelial cells while establishing a pro-tumorigenic microenvironment via NF-κB-mediated inflammatory pathways [39]. ERBB2 is expressed at low levels in normal tissue cells and at high levels in intestinal injury cells [40]. Additionally, ERBB2 mutations affect the ganglionic regions regulating the intestinal tract, causing abnormalities in the nervous system. These abnormalities result in complex intestinal motility disorders, such as slowed peristalsis, constipation, and diarrhea [41].

In our analysis of the enrichment pathways, we found that the enterotoxicity induced by PPDs and PPDQs is predominantly associated with several key pathways, such as the cancer pathway, MAPK signaling pathway, and calcium signaling pathway. The cancer pathway constitutes an integrated collection of signaling pathways that primarily delineate the molecular mechanisms and regulatory networks closely linked to cancer initiation and progression. It was previously shown to be differentially enriched in bowel cancer and crucial to bowel cancer development [42]. Considering the regulatory roles of cancer-related KEGG pathways and key targets, such as the influence of SRC on bowel cancer cells, we propose that PPDs and PPDQs may significantly affect both the incidence and metastasis of bowel cancer. Additionally, our enrichment analysis suggests that the enterotoxicity caused by PPDs and PPDQs is also linked to the MAPK signaling pathway. It is essential for regulating various cellular processes, including cell proliferation, differentiation, survival, and apoptosis. Notably, a number of inflammatory illnesses and malignant tumors exhibit persistent activation of the MAPK signaling pathway, which is strongly linked to the development, course, and outcome of the disease. Gene alterations, receptor overexpression, or aberrant stimulation in the tumor microenvironment are the most common causes of overactivation of the MAPK pathway in malignant tumors. In addition to encouraging tumor cell migration, invasion, and proliferation, this aberrant activation strengthens the tumor cell resistance to apoptosis, which helps them avoid host immune surveillance and withstand therapy [43]. Persistent activation of the MAPK pathway can drive the development of intestinal tumors. On one hand, it promotes proliferation and inhibits apoptosis through ERK/JNK-mediated activation of c-Myc/AP-1; on the other hand, it induces immune evasion by facilitating IL-10/TGF-β secretion and upregulating PD-L1. Additionally, this pathway synergizes with WNT/β-catenin to maintain tumor stemness, thereby playing a central role in inflammation-associated intestinal cancer [44]. Additionally, previous studies indicate the MAPK pathway mediates hyperproliferation and apoptosis of intestinal cancer cells. When the ERK/MAPK signaling pathway is blocked with inhibitor U0126, the growth and migration of colon cancer cells are inhibited [45]. Regarding the calcium signaling pathway, ca^2+^, as an important secondary messenger, it influences various physiological responses of cells by regulating fluctuations in intracellular calcium concentration, including cell growth, proliferation, and apoptosis, and calcium signaling is strongly associated with cancer through a novel integration of network toxicology and molecular docking [46]. According to previous studies, colorectal cancer is linked to a transient receptor potential channel in the calcium signaling pathway called TRPM8, and ingredients of the calcium signaling system are rearranged, altered, or deregulated in cancer [47]. These findings lead us to the conclusion that PPDs and PPDQs can cause bowel cancer via a variety of routes, such as the MAPK signaling pathway, calcium signaling pathway, and others.

Our study advances environmental toxicology, which overcomes traditional limitations of single-target analyses by prioritizing 30 hub targets from 182 candidates and validating IPPD-Q’s pan-binding capability (ΔG < −5 kcal·mol^−1^ for SRC/EGFR/ CASP3/MTOR). Among them, our PPI network analysis revealed that the 30 hub targets do not act in isolation but form interdependent signaling clusters (Figure 3). As one of the most ancient and extensively studied proto-oncogenes, SRC serves as a central signaling hub regulating critical cellular processes including proliferation, survival, inflammatory responses, and stress adaptation [48]. Emerging evidence highlights its crucial role in PPD/PPDQ-mediated intestinal toxicity. Molecular docking analyses reveal strong binding affinity between PPDQs (e.g., IPPD-Q) and SRC (ΔG = −6.7 kcal/mol), suggesting these compounds may mimic endogenous ligands to constitutively activate SRC kinase activity. Furthermore, SRC orchestrates multiple oncogenic pathways—including MAPK, PI3K/AKT, and STAT3 signaling cascades—to drive aberrant intestinal epithelial proliferation while suppressing apoptosis [49]. This mechanistic framework establishes SRC as both a molecular mediator and potential therapeutic target in PPD/PPDQ-associated intestinal inflammation and carcinogenesis. Additionally, SRC and EGFR exhibit direct physical interactions, suggesting a potential kinase cascade where SRC phosphorylates EGFR to amplify MAPK signaling [50]. Concurrently, CASP3 may counterbalance this proliferative drive by inducing apoptosis, yet its activity is suppressed by MTOR-mediated survival pathways. This delicate equilibrium mirrors the regulation observed in gut homeostasis, where pollutants like PPDQs tip the balance toward carcinogenesis. Notably, IPPD-Q’s pan-binding capability (ΔG < −5 kcal·mol^−1^ for all four targets) indicates its role as a master disruptor of this network. Furthermore, we revealed systemic crosstalk between kinase signaling and apoptotic pathways. Specifically, PPDs/PPDQs disrupt the MAPK–cancer–calcium signaling axis, where calcium influx via TRPM8 synergizes with EGFR/SRC to amplify proliferative signals, while calcium overload concurrently activates CASP3-dependent apoptosis: a bistable imbalance driving inflammation and carcinogenesis, as evidenced in zebrafish models. These findings underscore immediate applications, such as prioritizing IPPD-Q/CPPD-Q for regulatory monitoring due to their multi-target potency and repurposing MAPK inhibitors (e.g., U0126) to mitigate gut toxicity. Additionally, SRC/EGFR overexpression in PPD-exposed populations could serve as an early biomarker for intestinal damage. While this study provides a systematic prediction of PPD/PPDQ-induced intestinal toxicity, several limitations should be acknowledged. Firstly, the docking results require re-docking validation and further verification through molecular dynamics simulations due to the inherent constraints of static molecular modeling. Secondly, the synergistic effects of co-exposure with other environmental pollutants remain unexplored. Future studies should validate the expression patterns of SRC/EGFR in intestinal tissues, employ zebrafish models to assess in vivo toxicity, and integrate multi-omics approaches (e.g., transcriptomics-metabolomics) to further elucidate the metabolic impacts induced by PPDQs.

## 5. Conclusions

This study thoroughly examined the potential enterotoxicity of PPDs and PPDQs using network toxicology and molecular docking analysis techniques. We found 182 potential targets linked to enterotoxicity caused by PPDs and PPDQs, and we further screened 30 core targets, including SRC, EGFR, CASP3, and MTOR, which may be key factors in inducing the toxic effects of PPD and PPDQ on the intestinal tract. By controlling the apoptosis and proliferation of bowel cancer cells and triggering inflammatory signaling pathways, PPDs and PPDQs may cause bowel cancer and enteric-related inflammation. This study offers a theoretical foundation for comprehending the molecular mechanism of enterotoxicity caused by PPDs and PPDQs, which is beneficial for preventing and treating associated illnesses.

## Figures and Tables

**Table 1:** Key targets filtered out of the PPI network.

Gene	Degree	Closeness Centrality	Betweenness Centrality	Topological Coefficlent
SRC	88	0.655677	0.10274	0.166128
EGFR	86	0.646209	0.070655	0.177292
CASP3	85	0.646209	0.067594	0.176605
MTOR	69	0.610921	0.05981	0.191718
ERBB2	69	0.606779	0.051657	0.192028
NFKB1	69	0.604729	0.045221	0.19494
MMP9	63	0.594684	0.040975	0.191118
PTGS2	60	0.588815	0.037339	0.188631
GSK3B	59	0.583061	0.033291	0.196352
PIK3CA	57	0.573717	0.029618	0.2106307
PRKACA	54	0.571884	0.028589	0.152588
PARP1	48	0.559375	0.027703	0.212075
MAPK14	48	0.557632	0.019497	0.217721
KDR	46	0.5559	0.019177	0.217391
ABL1	46	0.549079	0.019049	0.223273
JAK2	45	0.549079	0.01485	0.238955
IGF1R	44	0.5474	0.014761	0.231476
MAPK8	42	0.5474	0.01471	0.21956
FYN	42	0.545731	0.014464	0.230064
PGR	41	0.545731	0.014268	0.231276
BRAF	41	0.544072	0.013927	0.231997
ICAM1	40	0.534328	0.01352	0.242438
NR3C1	39	0.532738	0.012948	0.212789
LYN	38	0.531157	0.012811	0.219864
PIK3CG	37	0.529585	0.012061	0.226415
PRKCA	37	0.528023	0.011536	0.233894
NTRK1	37	0.524926	0.01141	0.240453
PIK3CD	37	0.5249268	0.011282	0.241419
SYK	36	0.523391	0.011099	0.260243
KIT	36	0.523391	0.011074	0.263979

**Table 2:** Top 10 compounds in PPDs and PPDQs median values from PPI network.

Serial Number	Chemical Components	Degree
1	IPPD-Q	73
2	CPPD-Q	68
3	DPPD-Q	67
4	6PPD-Q	65
5	77PD	21
6	7PPD	19
7	44PD	16
8	6PPD	9
9	CPPD	5
10	DNPD	3

**Table 3:** PDB ID of the selected proteins.

Gene	PDB ID
SRC	1A07
EGFR	1M14
CASP3	1GFW
MTOR	1AUE

## Data Availability

The data that support the findings of this study will be available in Figshare at [https://doi.org/10.6084/m9.figshare.28852250] (accessed on 24 April 2025).
